# Strategies for discovery and validation of methylated and hydroxymethylated DNA biomarkers

**DOI:** 10.1002/cam4.22

**Published:** 2012-09-14

**Authors:** Ekaterina Olkhov-Mitsel, Bharati Bapat

**Affiliations:** 1 Samuel Lunenfeld Research Institute, Mount Sinai HospitalToronto, Ontario, Canada; 2 Department of Laboratory Medicine and Pathobiology, University of TorontoToronto, Ontario, Canada; 3 Department of Pathology, University Health Network, University of TorontoToronto, Ontario, Canada

**Keywords:** Affinity-based methylation analysis, bisulfite modification, hydroxymethylation, methylation-sensitive restriction enzymes, microarrays, next-generation sequencing

## Abstract

DNA methylation, consisting of the addition of a methyl group at the fifth-position of cytosine in a CpG dinucleotide, is one of the most well-studied epigenetic mechanisms in mammals with important functions in normal and disease biology. Disease-specific aberrant DNA methylation is a well-recognized hallmark of many complex diseases. Accordingly, various studies have focused on characterizing unique DNA methylation marks associated with distinct stages of disease development as they may serve as useful biomarkers for diagnosis, prognosis, prediction of response to therapy, or disease monitoring. Recently, novel CpG dinucleotide modifications with potential regulatory roles such as 5-hydroxymethylcytosine, 5-formylcytosine, and 5-carboxylcytosine have been described. These potential epigenetic marks cannot be distinguished from 5-methylcytosine by many current strategies and may potentially compromise assessment and interpretation of methylation data. A large number of strategies have been described for the discovery and validation of DNA methylation-based biomarkers, each with its own advantages and limitations. These strategies can be classified into three main categories: restriction enzyme digestion, affinity-based analysis, and bisulfite modification. In general, candidate biomarkers are discovered using large-scale, genome-wide, methylation sequencing, and/or microarray-based profiling strategies. Following discovery, biomarker performance is validated in large independent cohorts using highly targeted locus-specific assays. There are still many challenges to the effective implementation of DNA methylation-based biomarkers. Emerging innovative methylation and hydroxymethylation detection strategies are focused on addressing these gaps in the field of epigenetics. The development of DNA methylation- and hydroxymethylation-based biomarkers is an exciting and rapidly evolving area of research that holds promise for potential applications in diverse clinical settings.

## Introduction

Epigenetics is the study of reversible, heritable mechanisms that regulate gene expression without altering the DNA sequence [1, 2]. DNA methylation is one of the most well-studied epigenetic mechanisms in mammals. It refers to the addition of a methyl group to the fifth carbon of a cytosine (5-mC) that precedes a guanine (CpG). Frequently, but not exclusively, CpG dinucleotides occur in CG-rich DNA stretches known as CpG islands (CGIs) [3]. CGIs are often clustered within control regions of a gene, such as the promoter regions, but also less commonly in other parts of the gene, including introns and exons [4]. Recently, methylation has also been shown to occur at “CGI shores,” regions of lower CpG density that lie in close proximity, but not within CGIs [5, 6]. DNA methylation has many diverse functions in normal cells including silencing of transposable elements, inactivation of viral sequences, maintenance of chromosomal integrity, X-chromosome inactivation, and transcriptional suppression of a large number of genes [7, 8]. In normal cells, methylation patterns are replicated with high fidelity during mitosis. However, it has been shown that these patterns can become altered during the course of aging and disease. Aberrant DNA methylation is a well-recognized hallmark of many complex diseases such as heart disease, diabetes, and neurological disorders, but has been most extensively studied in cancer. Accordingly, various investigative teams have focused on characterizing unique DNA methylation “signatures” associated with pathogenesis as they may serve as useful biomarkers for diagnosis, prognosis, disease monitoring, or prediction of response to therapy [9].

DNA methylation biomarkers offer several significant advantages over expression-based markers. For instance, they are readily amplifiable and easily detectable using polymerase chain reaction (PCR)-based approaches even if alterations are present only in a limited number of cells [10]. DNA methylation is a highly stable marker that can be readily detected in a great variety of samples collected in a minimally invasive manner such as saliva, plasma, serum, urine, semen, and stool [11]. Furthermore, disease-specific DNA hypermethylation is a positively detectable signal. Despite these advantages, shortcomings in DNA methylation detection technologies including issues with assay sensitivity, specificity, accuracy, and data interpretation are confounding the discovery and development of effective clinical biomarkers. One limitation of DNA methylation analysis techniques is inability to differentiate heterogeneous methylation patterns in different cells present within samples [12]. Therefore, advances in technology that allow for analysis of a single DNA strand from a single cell will help point toward better biomarkers.

Another limitation of many current methodologies is the inability to distinguish between 5-mC and other novel structurally similar DNA modifications that have been recently discovered in mammalian DNA including 5-hydroxymethylcytosine (5-hmC), 5-formylcytosine (5-fC), and 5-carboxylcytosine (5-caC) [13]. 5-hmC has been recently discovered to be generated by hydroxylation of 5-mC by a group of enzymes of the 10–11 translocation (TET) proteins and is now considered to be “the sixth base” of the genome of higher organisms [14–16]. This raises the possibility that 5-hmC may act as an intermediate epigenetic state associated with changes in DNA methylation and transcriptional regulation during development, normal, and disease states [14, 16, 17]. Studies have shown a correlation between 5-hmC and gene expression, suggesting a regulatory role for 5-hmC [18–20]. Furthermore, it was recently shown that 5-hmC is significantly decreased in multiple human cancers and cancer mouse models, opening exciting opportunities to explore new types of epigenetic biomarkers [17, 21]. To address this, innovative 5-hmC detection methods are being developed to allow for specific and/or simultaneous detection of 5-mC and 5-hmC. However, further research is necessary in the area of 5-fC and 5-caC detection strategies. Improvements in technology may lead to the development of novel epigenetic biomarkers that will enhance our understanding of the molecular biology of diseases.

**Figure 1:**
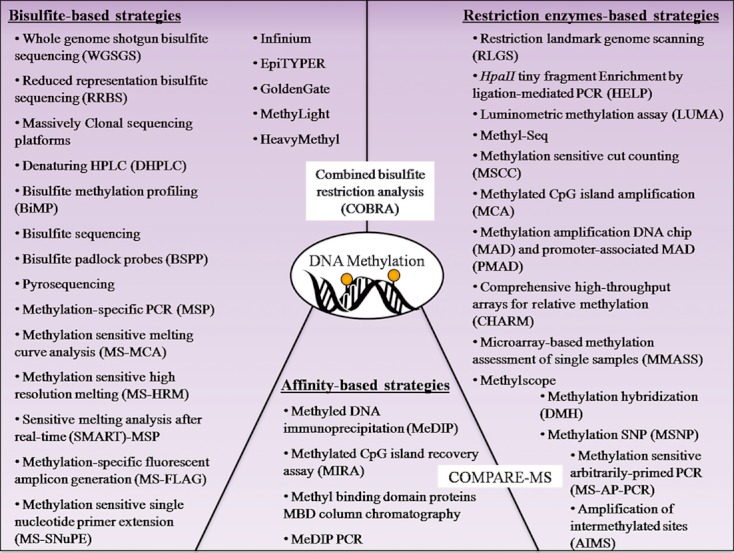
Main strategies for DNA methylation analysis classified into three categories: restriction enzymes-based, affinity-based, and bisulfite-based strategies. The COBRA approach has been placed between bisulfate-based and restriction enzymes-based strategies, while the COMPARE-MS approach has been placed between restriction enzymes-based and affinity-based strategies because these combine two approaches. COMPARE-MS, combination of methylated-DNA precipitation and methylation-sensitive restriction enzymes.

This review is divided into two parts that cover existing and emerging strategies applied to (A) discovery and (B) validation of DNA methylation-based biomarkers and describes their major advantages and limitations (Fig. 1). Particularly, more recent strategies that have not been previously reviewed in the literature are described in more detail. Part A gives an overview of the large-scale, genome-wide, epigenetic profiling platforms used for candidate biomarker discovery. These platforms can be used to compare methylation profiles among cell lines, healthy samples, and disease samples to find disease-related alterations. Tables 1 and 2 provide an overview of these genome-wide methylation analysis strategies and their applications to sequencing (Table 1) and microarray (Table 2) platforms and their significant advantages and limitations. Part B gives an overview of highly targeted locus-specific assays used for validation of biomarker performance in large independent cohorts. Table 3 provides an overview of locus-specific assays developed for analysis of a few loci across numerous samples and their advantages and limitations, whereas Table 4 presents information on the sensitivity and DNA quality requirements of each strategy. Additionally, studies examining the effect of hydroxymethylation on the outcome of methylation marker analyses and novel detection strategies specific to 5-hmC are described.

**Table 1:** Overview of sequencing strategies for global methylation analysis

Strategy	Description	Advantages	Limitations	References
Restriction enzyme digestion				
RLGS	DNA is digested with methylation-sensitive enzymes such as *Not*I or *Asc*I	Methylation profiles are reproducible and quantitative	Labor intensive	[22, 23]
			Radioactive material required	
			Difficulties in reaction product identification	
HELP-seq	DNA is digested with *Hpa*II or *Msp*I	Methodology is simple and cost-effective	Require DNA of high quantity, purity, and integrity	[26]
Methyl-Seq	DNA is digested with *Hpa*II or *Msp*I	Enzyme digestion site occurs frequently in CGI	Not well suited to distinguish moderately and weakly methylated fragments	[27]
LUMA	DNA undergoes digestion with *Eco*RI+*Hpa*II or *Eco*RI+*Msp*I and polymerase extension assay by Pyrosequencing	Quantitative	Limited to restriction enzymes digestion sites	[28, 29]
		Requires less DNA quantity than other restriction enzymes based methods		
MSCC	DNA is digested with *Hpa*II and *Mme*I	Allow for analysis of extremely CpG-rich CGI	Require DNA of high quantity, purity, and integrity	[30]
MCA-seq	DNA is digested with *Sma*I and *Xma*I			[31]
			Not well suited to distinguish moderately and weakly methylated fragments	
Affinity-based methylation analysis				
MeDIP-Seq	Single-stranded DNA is immunoprecipitated with anti-5-methylcytosine antibodies	Allow for rapid and specific assessment of the mean methylation levels of	Requires DNA to be single-stranded	[53]
		Large DNA regions	Limited by the quality and specificity of the antibody	
		Reagents involved are commercially available and easy to use.	Sequence bias	
MIRA	Utilizes MBD2b/MBD3L1 protein complex		No information on distinct CpG dinucleotides	[57]
			Sequence bias	
Bisulfite modification				
WGSGS	Whole genome shotgun sequencing of bisulfite-modified DNA	Allows methylation analysis of every CpG in the Genome	Cost of sequencing the entire human genome is currently too expensive	[72, 73]
RRBS	bisulfite-modified DNA is digested with *Bgl*II or *Msp*I	Less costly than other bisulfite-based methods	Limited to restriction enzymes digestion sites	[76, 77]
DHPLC	bisulfite-modified DNA is passed through HPLC under partially denaturing conditions	High sample throughput	Requires expensive equipment and extensive optimization	[78]

RLGS, restriction landmark genome scanning; HELP, *Hpa*II tiny fragment enrichment by ligation-mediated PCR; LUMA, luminometric methylation assay; MSCC, methylation-sensitive cut counting; MCA, methylated CpG island amplification; MeDIP, methyled DNA immunoprecipitation; CGI, CpG island; MIRA, methylated CpG island recovery assay; WGSGS, whole genome shotgun bisulfite sequencing; RRBS, reduced representation bisulfite sequencing; DHPLC, denaturing HPLC.

**Table 2:** Overview of microarray strategies for global methylation analysis

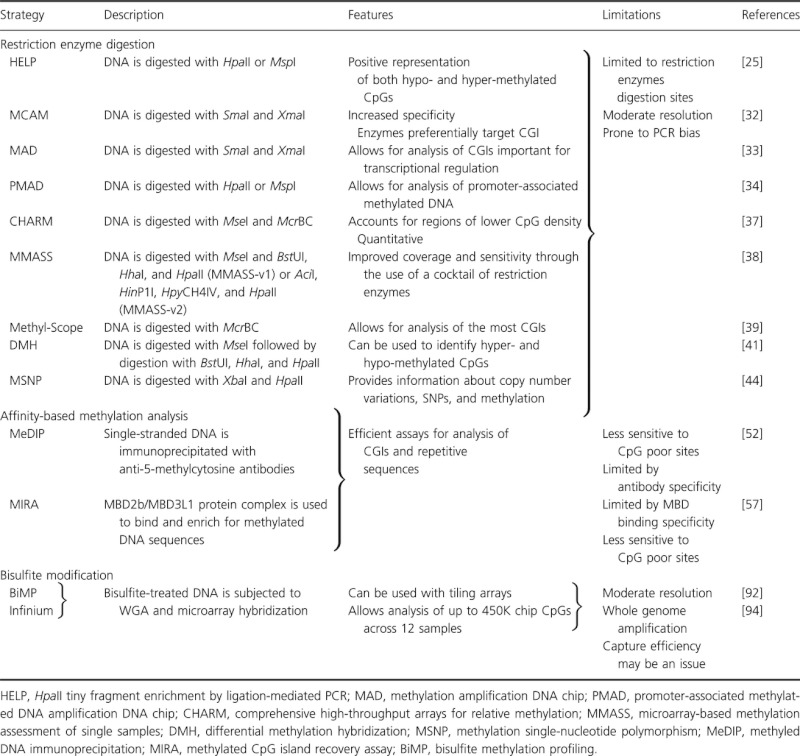

**Table 3:** Overview of locus-specific strategies for methylation analysis

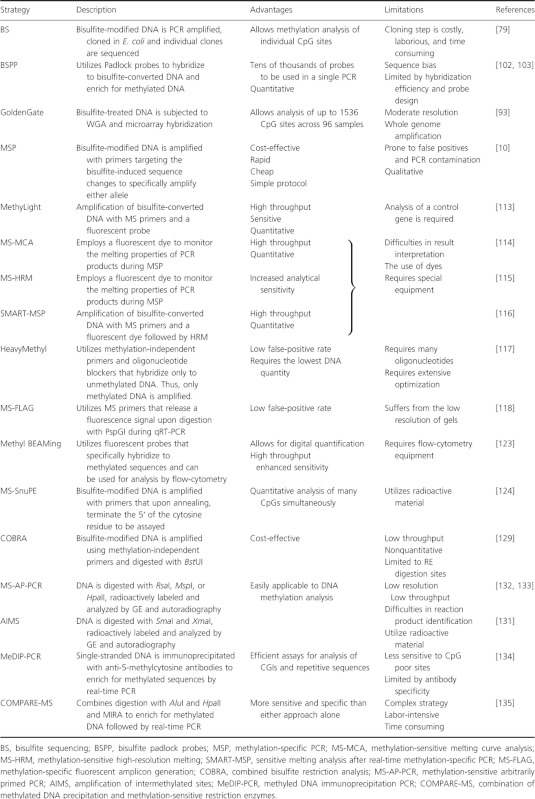

**Table 4:** Comparison of the sensitivity and DNA quality requirements for various DNA methylation analysis strategies

Strategy	Sensitivity^1^	DNA quality requirement
RLGS	Low	High-quality input DNA is required
HELP	Sensitivity is CpG content dependent	High-quality input DNA is required
	There is higher sensitivity for lower CpG density regions	
Methyl-Seq	High	High-quality input DNA is required
LUMA	High	High-quality input DNA is required
MSCC	High	High-quality input DNA is required
MCA-seq	High	High-quality input DNA is required
MeDIP-Seq	Sensitivity is CpG content dependent	High-quality input DNA is required
	There is higher sensitivity for high CpG density regions	
MIRA	High	High-quality input DNA is required
WGSGS	High	High-quality input DNA is required
RRBS	High	High-quality input DNA is required
BSPP	Sensitivity varies with protocol	High-quality input DNA is required
DHPLC	Sensitivity depends on temperature optimization	Modest quality input DNA is required
CHARM	Medium	High-quality input DNA is required
MMASS	Medium	High-quality input DNA is required
Methyl-Scope	High	High-quality input DNA is required
DMH	Medium	High-quality input DNA is required
BiMP	Medium	High-quality input DNA is required
GoldenGate	High	Modest quality input DNA can be analyzed
Infinium	High	High-quality input DNA is required
BS	Medium	Moderate-quality input DNA can be analyzed
	Sensitivity varies with number of clones sequenced	
Pyrosequencing	Medium	Moderate-quality input DNA can be analyzed
MSP	High	Modest quality input DNA, for example, extracted from formalin fixed paraffin-embedded tissues can be analyzed
MethyLight	High	Minute amounts of modest quality DNA are required
MS-MCA	Medium	Modest quality input DNA can be analyzed
MS-HRM	High	Modest quality input DNA can be analyzed
SMART-MSP	High	Modest quality input DNA can be analyzed
HeavyMethyl	High	Modest quality input DNA can be analyzed
MS-FLAG	High	Modest quality input DNA can be analyzed
Methyl BEAMing	High	Modest quality input DNA can be analyzed
MS-SnuPE	Medium	Moderate-quality input DNA can be analyzed
COBRA	Medium	Modest quality input DNA can be analyzed
MS-AP-PCR	Low	Moderate-quality input DNA can be analyzed
AIMS	Low	Moderate-quality input DNA can be analyzed
MeDIP-PCR	Low	Moderate-quality input DNA can be analyzed
COMPARE-MS	High	Modest quality input DNA can be analyzed

RLGS, restriction landmark genome scanning; HELP, *Hpa*II tiny fragment enrichment by ligation-mediated PCR; LUMA, luminometric methylation assay; MSCC, methylation-sensitive cut counting; MCA, methylated CpG island amplification; MeDIP, methyled DNA immunoprecipitation; MIRA, methylated CpG island recovery assay; WGSGS, whole genome shotgun bisulfite sequencing; RRBS, reduced representation bisulfite sequencing; BSPP, bisulfite padlock probes; MSP, methylation-specific PCR; DHPLC, denaturing HPLC; CHARM, comprehensive high-throughput arrays for relative methylation; MMASS, microarray-based methylation assessment of single samples; DMH, differential methylation hybridization; BiMP, bisulfite methylation profiling; BS, bisulfite sequencing; MSP, methylation-specific PCR; MS-MCA, methylation-sensitive melting curve analysis; MS-HRM, methylation-sensitive high-resolution melting; SMART-MSP, sensitive melting analysis after real-time methylation-specific PCR; MS-FLAG, methylation-specific fluorescent amplicon generation; COBRA, combined bisulfite restriction analysis; MS-AP-PCR, methylation-sensitive arbitrarily primed PCR; AIMS, amplification of intermethylated sites; MeDIP-PCR, methyled DNA immunoprecipitation PCR; COMPARE-MS, combination of methylated DNA precipitation and methylation-sensitive restriction enzymes.

1 Sensitivity is dependent on the specific assay and parameters such as the concentration and quality of input DNA and PCR conditions. For this reason, we have not defined absolute values for this parameter.

## Discovery of Novel DNA Methylation Biomarkers

Over the past few decades, there have been an increasing number of approaches devoted to generating genome-wide methylation profiles and aberrant methylation signatures, each with its own advantages, disadvantages, and areas of applicability. As DNA methylation information is lost during PCR amplification, the majority of techniques rely on methylation-dependent treatment of DNA prior to amplification. These assays can be classified into three main categories: restriction enzyme (RE) digestion, affinity-based analysis, and bisulfite modification. The combination of these three approaches with sequencing and microarray-based platforms has given rise to a wide range of techniques for global DNA methylation analysis.

Global approaches to DNA methylation analysis are being widely used to generate genome-wide methylation profiles because they offer a number of advantages. In general, these approaches are high-throughput strategies with regard to the number of loci that can be analyzed at one time. In particular, sequencing platforms provide quantitative information about the methylation status of every CpG and allow for the analysis of methylation in repeat sequences and rare methylation variants, which is difficult to do using microarrays. Another advantage of sequencing approaches is that they can be used to analyze DNA methylation of regions with no prior knowledge of the sequence. The main weaknesses of sequencing strategies are library bias, cost, availability, and difficulties in data management and analysis, although the cost of massive sequencing technologies is rapidly decreasing. DNA methylation profiling using high-density microarrays is another commonly used method to identify broad differences between groups of samples. They are less time consuming, less labor intensive, and less costly than sequencing. In addition, microarrays allow for simultaneous analysis of a larger number of samples with a wider CGI coverage. Nevertheless, microarray analyses lack reliable quantitation and are limited by probe design, hybridization efficiency, and hybridization artifacts.

### Restriction enzyme digestion

**Figure 2:**
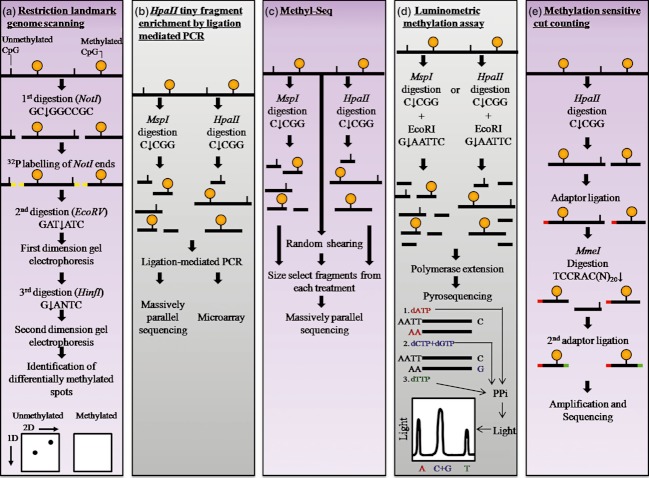
A panel of methylation-sensitive restriction enzyme-based strategies for DNA methylation analysis including (a) restriction landmark genomic scanning (RLGS), (b) *Hpa*II tiny fragment enrichment by ligation-mediated PCR (HELP), (c) Methyl-Seq, (d) luminometric methylation assay (LUMA), and (e) methylation-sensitive cut counting (MSCC). (a) In RLGS, genomic DNA is digested with a methylation-sensitive enzyme such as *Not*I, radioactive nucleotides are incorporated into the *Not*I half-sites, and size-fractionation is achieved using gel electrophoresis. The digestion products are further digested with two more restriction enzymes and the fragments are separated by two-dimensional electrophoresis. On the gel, unmethylated DNA is indicated by a spot on the gel, whereas methylated DNA has no corresponding spot on the gel. (b) In HELP, DNA is digested with the methylation-sensitive enzyme *Hpa*II. In parallel, a second aliquot of DNA is digested with the methylation-insensitive isoschizomer, *Msp*I. The digestion products are PCR amplified and analyzed by microarrays or sequencing. (c) In Methyl-Seq, DNA is either digested with *Msp*I, *Hpa*II, or randomly sheared. The digestion products are size fractioned and the selected fragments are sequenced. (d) In LUMA, DNA is digested with *Hpa*II or *Msp*I followed by digestion with EcoRI, bioluminometric polymerase extension, and pyrosequencing. (e) In MSCC, DNA is digested by *Hpa*II, followed by adaptor ligation, *Mme*I digestion, second adaptor ligation, PCR amplification and sequencing.

**Figure 3:**
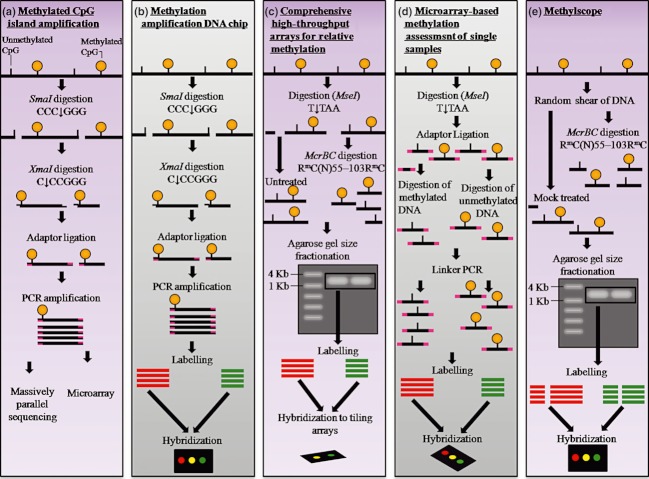
A panel of methylation-dependent and methylation-sensitive restriction enzyme-based strategies for DNA methylation analysis including (a) methylated CpG island amplification (MCA), (b) methylation amplification DNA chip (MAD), (c) comprehensive high-throughput arrays for relative methylation (CHARM), (d) microarray-based methylation assessment of single samples (MMASS), and (e) MethylScope. (a) In MCA, genomic DNA undergoes digestion with *Sma*I followed by *Xma*I, adaptor ligation, and PCR amplification. Methylation is then assessed by microarrays or sequencing. (b) In MAD, DNA digested with *Sma*I and *Xma*I is PCR amplified, labeled, and cohybridized to microarrays specifically developed for CpG island methylation analysis. (c) In CHARM, *Mse*I digested DNA is separated into two: one-half is digested with *Mcr*BC to cut methylated sequences and the other is undigested. Digestion products are size fractioned by gel electrophoresis, and fragments of selected size are purified from the gel, labeled, and cohybridized to tiling arrays. (d) In MMASS, *Mse*I-digested DNA is separated into two: one-half is digested with *Mcr*BC to cut methylated sequences and the other is cut with methylation-sensitive enzymes to cut unmethylated sequences. The fragments are then PCR amplified, labeled, and cohybridized to microarrays. (e) In MethylScope, randomly sheared DNA is separated to aliquots: one is digested with *Mcr*BC, while the other is untreated. Digestion products are size fractioned by gel electrophoresis, and fragments of selected size are purified from the gel, labeled, and cohybridized to tiling arrays.

Restriction enzyme-based methods exploit the property of methylation-sensitive enzymes which only digest unmethylated DNA and methylation-dependent enzymes which only cut methylated DNA. These enzymes are used to enrich for methylated or unmethylated sequences and provide a read-out of DNA methylation. Restriction landmark genome scanning (RLGS) was the first reliable RE-based technique for global DNA methylation profiling and has been previously reviewed in detail by Smiraglia et al. among others (Fig. 2) [22–24]. However, the use of RLGS is decreasing as it involves the use of radioactive materials and gel electrophoresis. Many techniques currently in use couple enzymatic methods to array-based analysis. One such technique is *Hpa*II tiny fragment enrichment by ligation-mediated PCR (HELP) which is based on digestion of high-molecular-weight genomic DNA with methylation-sensitive *Hpa*II (Fig. 2) [25]. In parallel, a second aliquot of DNA is digested with the methylation-insensitive isoschizomer, *Msp*I, which digests the same cleavage site irrespective of methylation status. Therefore, sequences present in *Msp*I but not in *Hpa*II libraries are derived from methylated regions. The *Msp*I library also serves as an internal control that allows for identification of spurious variables that can affect *Hpa*II digestion. These include absence of CpG sites in restriction site, mutations, copy number variations, and technical failure. Furthermore, the use of an internal reference allows for detection of spurious differential effects specific to the *Hpa*II enzyme. The HELP assay has been combined with massively parallel sequencing (HELP-Seq) and/or array-based platforms [25, 26]. Other examples of approaches based on *Hpa*II and *Msp*I digestion are methyl-Seq and luminometric methylation assay (LUMA) (Fig. 2) [27–29]. In methyl-Seq, following digestion with *Msp*I and *Hpa*II, genomic DNA fragments are subjected to size selection to enrich for CpG-containing regions and the selected fragments are sequenced on a next-generation sequencing platform. In LUMA, genomic DNA is cleaved by *Hpa*II or *Msp*I followed by a bioluminometric polymerase extension and pyrosequencing to quantify the extent of RE cleavage and thus methylation levels. To enable normalization between runs and for DNA input, *Eco*RI is included in all reactions. The above approaches rely on *Msp*I digestion to create a control library. Alternatively, in methods such as methylation-sensitive cut counting (MSCC), genomic DNA is only digested with *Hpa*II followed by deep sequencing (Fig. 2) [30]. The number of times a given site is observed during sequencing then serves as indication of methylation level. Sites represented many times during sequencing are inferred to have low methylation while sites with no reads have high methylation levels. Besides *Hpa*II*/Msp*I, another enzyme pair commonly used in methylation analyses is *Sma*I (methylation sensitive) and *Xma*I (methylation insensitive). One method utilizing these enzymes is methylated CGI amplification (MCA) [31]. This method employs *Sma*I to generate blunt end fragments and to eliminate unmethylated sites (Fig. 3). Next, DNA is further digested with *Xma*I to create sticky ends and leave overhangs in methylated sites. Methylated fragments are then adaptor ligated and PCR enriched. The resulting amplicons are either sequenced (MCA-Seq) or differentially labeled and cohybridized to a microarray (MCAM) [31, 32]. Another strategy that utilizes the *Sma*I and *Xma*I enzymes is methylation amplification DNA chip (MAD) (Fig. 3) [33]. More recently, MAD was modified to develop the promoter-associated methylated DNA amplification DNA chip (PMAD) assay which incorporates the *Hpa*II and *Msp*I enzymes [34]. Both techniques have been previously reviewed by Huang et al. [35].

An alternative to using methylation-sensitive enzymes is to use methylation-dependent enzymes as *Mcr*BC. This enzyme recognizes closely spaced methylated cytosines and so has the capacity to digest densely methylated regions of DNA [36]. One technique that utilizes this enzyme is comprehensive high-throughput arrays for relative methylation (CHARM) [37]. The initial step in this method is digestion with RE such as *Mse*I to shear DNA (Fig. 3). The recognition site of this enzyme rarely occurs in GC-rich-regions; thus; most CGIs remain intact. This is followed by the division of DNA into two fractions: one treated with *Mcr*BC and the other untreated. The *Mcr*BC digested and untreated DNA is size-fractionated, differentially labeled, and cohybridized to a microarray. The ratio of hybridization intensities between treated and untreated DNA provides a measure of DNA methylation. Other techniques that utilize the *Mcr*BC enzyme are microarray-based methylation assessment of single samples (MMASS, Fig. 3), which has been reviewed by Huang et al., and MethylScope [35, 38–40]. With the MethylScope strategy DNA is sheared and divided into two fractions, one of which is digested with *Mcr*BC (Fig. 3). The fragments are then fractionated by electrophoresis and fragment larger than 1 kb are purified, labeled with different dyes for the *Mcr*BC digested and undigested fractions, and cohybridized to genomic-tiling microarrays.

**Figure 4:**
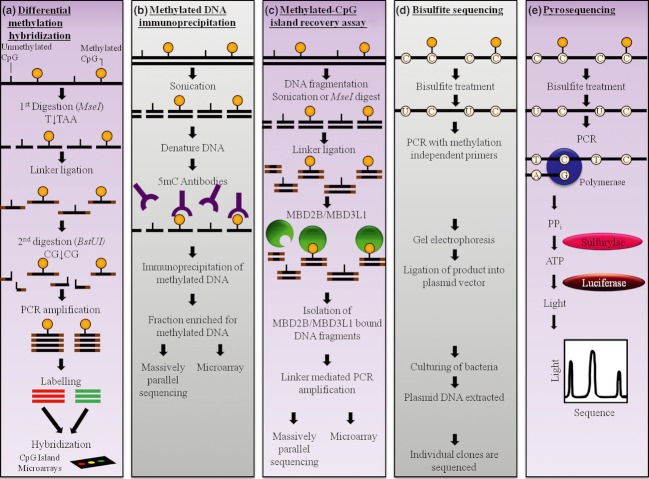
A panel of strategies for DNA methylation analysis including (a) differential methylation hybridization (DMH), (b) methyled DNA immunoprecipitation (MeDIP), (c) methylated CpG island recovery assay (MIRA), (d) bisulfite sequencing, and (e) pyrosequencing. (a) In DMH, genomic DNA is fragmented with a methylation-independent restriction enzyme and undergoes adaptor ligation. Next, DNA is digested with the methylation-sensitive enzyme *Bst*UI, PCR amplified, labeled, and cohybridized to CpG island microarrays. (b) In MeDIP, DNA is sheared through sonication, denatured, and immunoprecipitated with antibody against 5-methylcytidine. Methylated DNA is then analyzed using microarrays or sequencing. (c) In MIRA, DNA sheared by sonication or *Mse*I digestion undergoes adaptor ligation followed by incubation with MBD2b/MBD3L1 proteins. The MIRA captured DNA is then PCR amplified and analyzed using microarrays or sequencing. (d) In bisulfite sequencing, bisulfite-treated DNA is PCR amplified with methylation-independent primers and size fractioned using gel electrophoresis. The purified PCR products are then cloned into *E. coli* and individual clones (usually 5–10) are sequenced. (e) In pyrosequencing, bisulfite-modified DNA is amplified with DNA polymerase and sequencing primers. As the complementary DNA strand is synthesized, PP_i_ is released and converted into ATP. The ATP provides the energy to form a luciferase–luciferin–AMP complex, which in the presence of oxygen results in the release of light in a proportional amount to the available ATP and thus PP_i_.

The advantage of using *Mcr*BC is its high sensitivity to densely methylated regions. Also, as it does not require a highly specific sequence motif, it cuts more frequently. One other advantage of this assay is that it does not require prior methylation information from a reference genome to serve as a control. Other variations of RE-based DNA methylation profiling methods include those that employ a combination of methylation-sensitive enzymes. One such technique is differential methylation hybridization (DMH) [41]. In this approach, DNA is digested using a combination of methylation-sensitive enzymes such as *Bst*UI, *Hha*I, and *Hpa*II (Fig. 4). DNA fragments then undergo linker ligation, PCR enrichment, and cohybridization to a microarray. We and others have successfully implemented this strategy. For example, in our laboratory, transforming growth factor β 2 (*TGFβ*2) and homeobox D3 (*HOXD*3) hypermethylation has been discovered as potential biomarkers of prostate cancer progression through a genome-wide DMH screening [42, 43].

An additional microarray platform that enables the measurement of single-nucleotide polymorphisms (SNPs), copy number, loss of heterozygosity (LOH), and DNA methylation simultaneously is methylation SNP (MSNP) [44, 45]. In this approach, DNA is first sheared with *Xba*I, a frequent cutting enzyme, for genomic library construction. Next, the DNA is digested with *Hpa*II to enrich for methylated fragments. This way one can check for (1) copy number variations in *Xba*I fragments, (2) SNPs in *Hpa*II cutting sites in *Xba*I fragments, and (3) methylation in *Hpa*II cutting sites. This approach has the obvious advantage of providing information about numerous features from one array.

RE-based genome-wide DNA methylation analysis is a potentially robust approach for genome-wide screening to identify frequently methylated CpGs. The methodology is relatively straightforward, rapid, and inexpensive and can be used to analyze thousands of CpGs in a single experiment. Some of the earliest studies to find disease-specific gene methylation events which have been proposed as biomarkers relied on RE digestion. For example, methylation of O(6)-methylguanine DNA methyltransferase (*MGMT*) in gliomas, π-class glutathione S-transferase (*GSTP*1) in prostate cancer, and mutL homolog 1 (*MLH*1) in colon cancer were discovered using this strategy [46–48]. However, as these approaches are based on RE, they are confined to recognition elements and can only interrogate a subset of methylation sites. Another limitation of enzymatic approaches is the inability to distinguish 5-mC and 5-hmC [49]. Methylation-dependent enzymes cleave both CpG modifications (methylation and hydroxymethylation), whereas methylation-sensitive enzymes are completely blocked by both modifications. Consequently, a proportion of genomic loci identified as “methylated” in these studies may actually be hydroxymethylated. To address this issue, new enzymatic approaches have been developed for specific detection of hydroxymethylated cytosines. These include, but are not limited to, enzymatic digestion of DNA followed by radioactive labeling of the 5-hmC and enzymatic glucosylation strategies which utilize *β*-glucosyltransferase to attach a glucose moiety to 5-hmC, protecting it from subsequent digestion with glucosyl-sensitive REs [14, 50]. Alternatively, other strategies employ 5-hmC-dependent enzymes such as PvuRts1I which selectively cleave 5-hmC-containing sequences [51]. The enriched 5-hmC fractions can then be analyzed by DNA microarrays, sequencing, or chromatography.

### Affinity-based methylation analysis

To circumvent the limitations of RE digest analysis, techniques that use affinity purification to enrich for methylated DNA can be utilized. Techniques used to capture methylated DNA sequences as methyled DNA immunoprecipitation (MeDIP) start with shearing DNA through sonication to produce random fragments [52]. The fragments are then denatured to produce single-stranded DNA and immunoprecipitated with one or more monoclonal anti-5-methylcytosine antibodies (Fig. 4). The collected DNA is enriched for methylated sequences and is then amplified and analyzed using sequencing (MeDIP-Seq) or microarray platforms [52, 53]. Recently, coupling of MeDIP with microarray platforms has been proven to be a successful strategy to map genome-wide DNA methylation patterns in *Arabidopsis thaliana* as well as human normal and transformed cells [52, 54, 55]. One major limitation of the method is that MeDIP requires DNA to be single-stranded which may be difficult to achieve in regions of high CpG content. MeDIP-based methods are also limited by the quality and specificity of the antibody. Moreover, enrichment efficiency is significantly lower in regions with low CpG content.

To avoid these problems, methods based on methyl binding domain proteins (MBDs) can be used. Such methods include methylated CGI recovery assay (MIRA), which utilizes MBD2 and MBD3, and MBD column chromatography which utilizes MBD2 or MeCP2 [56–58]. In MIRA, DNA is sheared with *Mse*I, linker ligated, and incubated with MBD2 and MBD3 bound to a sepharose matrix that binds to methylated DNA with high specificity (Fig. 4). The MIRA captured DNA is PCR amplified, labeled, and cohybridized to CGI microarrays. Affinity-based methods allow for rapid and specific assessment of the mean methylation levels of large DNA regions. The reagents involved are commercially available and easy to use. However, the methods require high-DNA input and do not yield information on distinct CpG dinucleotides. Moreover, MBD or antibody interaction with DNA is affected by surrounding sequences and methylation density. Therefore, repeat sequences are sometimes overrepresented in affinity-based analysis.

Furthermore, it has been shown that affinity-based methylation strategies that utilize MBDs or anti-5-mC antibodies are specific and do not bind 5-hmC [59, 60]. Therefore, anti-5-hmC antibodies were developed for hydroxymethylation-specific analyses and can be used in the abovementioned strategies replacing anti-5-mC antibodies [19]. Anti-5-hmC-specific antibodies can also be used in combination with dot blots or immunohistochemical platforms to detect 5-hmC in cells and tissues [17, 61, 62]. Alternatively, numerous strategies that involve chemical labeling of 5-hmC (e.g., biotin or sulfonate) followed by affinity-based purification with specific antibodies have been developed [63, 64]. One such approach makes use of enzymatic glucosylation of 5-hmC followed by selective pull down using J-binding protein 1 coupled to magnetic beads [65].

### Bisulfite modification

The principle of sodium bisulfite modification is based on the differential reaction of methylated and unmethylated cytosines with the reagent, such that following bisulfite treatment, only unmethylated cytosines are converted into uracils [66]. The conversion can then be detected using a variety of methods combined with sequencing and/or microarray platforms. Bisulfite treatment-based strategies of methylation analysis surpass almost every other methodology, thereby becoming the most widely accepted and most widely used approaches. The advantages of this methodology include quantitative DNA methylation analysis almost anywhere in the genome, single CpG resolution, and detection of strand-specific methylation. However, the conversion process results in significant DNA degradation and reduced sequence complexity. This poses certain challenges for sequencing and array platforms. Moreover, methods relying on bisulfite conversion and sequencing also require extensive bioinformatics for base calling, sequence alignment, and statistical analysis. Additionally, as bisulfite analysis depends on the complete conversion of unmethylated cytosines to uracil, incomplete or inappropriate conversion will be erroneously interpreted. Studies have also shown that sodium bisulfite reacts with 5-hmC to yield a distinct adduct, cytosine 5-methylenesulfonate which does not undergo conversion to a deaminated cytosine [49, 60, 67]. Some have suggested that 5-methylenesulfonate may stall or block Taq polymerase in subsequent amplification reactions [67]. However, it has been shown that bisulfite-treated DNA templates containing 5-hmC can be efficiently amplified [49]. As a result, following bisulfite conversion, 5-hmC is indistinguishable from 5-mC, implying that a proportion of genomic loci previously identified as methylated may actually be hydroxymethylated.

Therefore, “oxidative bisulfite” sequencing (oxBS-Seq) approach has been recently developed [68]. In this approach, 5-hmC undergoes specific oxidation to 5-fC using potassium perruthenate. Next, during bisulfite conversion, 5-fC is converted to uracil allowing for specific mapping of 5-mC sites. Furthermore, 5-hmC mapping can be achieved by subtraction of oxBS-Seq from a BS-Seq readout.

Alternatively, bisulfite-independent strategies involving alternative chemical pretreatments of DNA have been recently developed for specific 5-hmC detection. One such approach is called glucosylation, periodate oxidation, biotinylation (GLIB) [69]. This strategy is based on initial glucosylation of 5-hmC followed by periodate oxidation and biotinylation. The hydroxymethylated DNA is then pulled down using the biotin-streptavidin system. Other related strategies have also been recently published using a custom-synthesized UDP-glucose analog (UDP-6-N3-glucose) or radioactively labeled UDP-[^3^H] glucose [63, 70]. Alternative chemical labeling strategies can be carried out by the addition of sulfur containing moieties, cysteamine, or selenocysteamine followed by direct detection or selective biotinylation [71]. The enriched 5-hydroxymethylated DNA can then be analyzed by microarrays or sequencing.

#### Sequencing-based methylation profiling

Whole genome shotgun bisulfite sequencing (WGSGS) provides a genome-wide methylation profile at single base-pair resolution and is therefore the most comprehensive methodology [72, 73]. It has recently been applied to generate a whole genome methylation profile of the *A. thaliana* genome [74, 75]. However, the human genome is much larger and the cost of sequencing is currently very expensive.

An alternative method, called reduced representation bisulfite sequencing (RRBS), enriches for CpG-rich regions using RE such as *Bgl*II or *Msp*I to reduce genome complexity and sequence redundancy [76, 77]. Next, DNA undergoes adaptor ligation, bisulfite modification, PCR enrichment, and finally sequencing. The data generated includes regions of the genome that are in close proximity to the RE's recognition site. That is simultaneously an advantage for bioinformatics analysis and a limitation for genome-wide methylation analysis.

An alternative approach to enrich for CpG-rich DNA is denaturing HPLC (DHPLC) [78]. This technique is based on the idea that following bisulfite treatment, amplicons that differ in methylation patterns have different G/C content resulting in different melting temperature, which in turn translates into different retention times in HPLC under partially denaturing conditions. The different DNA fractions are then sequenced to identify methylation profiles. The advantages of this technique are that it is simple, cost-effective, and rapid. However, it requires relatively high DNA quantities and has limited sensitivity, especially when analyzing tissue samples.

#### Massively parallel clonal DNA sequencing platforms

Sequencing-based methylation analyses initially relied on Sanger sequencing [79]. However, it is too costly, inefficient, and time consuming to sequence the entire human genome. Therefore, a variety of sequencing platforms have been developed and applied to DNA methylation analysis. These include next-generation sequencing (NGS) and single-molecule sequencing. The development of NGS platforms enables sequencing and mapping of millions of DNA fragments in parallel, thus significantly increasing throughput and decreasing cost per base thereby providing new opportunities for comprehensive, highly sensitive, genome-wide mapping of methylation sites at a more affordable price [80]. These methodologies are gradually replacing conventional sequencing. The three main NGS platforms currently used are Roche 454 sequencing (Branford, Connecticut), Applied Biosystems SOLiD™ (Carlsbad, California), and Illumina Solexa; genome analyzer (San Diego, California) [81–83]. Other NGS platforms also available are Polonator (Salem, New Hampshire) and Helicos Heliscope™ (Cambridge, Massachusetts) [84, 85]. Roche 454 sequencing was the first commercially available NGS platform. In this approach, clonal amplification of library fragments bound on beads is achieved by single-molecule emulsion PCR with amplicons captured onto the surface of beads. Individual beads are then sequenced by pyrosequencing. Roche 454 can generate up to one million reads per run at read lengths of up to 1 kbp (http://www.454.com/). It provides the fastest time per run and longest read length compared with other NGS platforms, offering several advantages for methylation analysis. Longer reads can be more easily and accurately aligned to the reference sequence and have a higher chance to cover SNPs and other genotyping information in the vicinity of CpGs. However, this strategy generates less reads per run resulting in higher cost of sequencing. Additionally, it has a higher error rate in calling homopolymeric stretches which may be a problem in bisulfite-modified DNA because it contains long stretches of A or T following conversion.

Similarly, Applied Biosystems SOLiD™ is also based on emulsion PCR to generate clonally amplified sequencing fragments with smaller beads attached to a solid surface and sequencing is achieved using sequencing by synthesis driven by a ligase. The Applied Biosystems SOLiD™ platform can generate up to 700 million reads per run at read lengths of up to 75 bp (http://www.appliedbiosystems.com). One advantageous feature of this platform is two base encoding in which each base position is examined twice; thus, miscalls can be more readily identified. Additionally, a new strategy, termed MethylSeq™, has been recently developed. In MethylSeq™, bisulfite-modified DNA is also amplified by microdroplet emulsion PCR using a primer library targeting a large number of genes. The resulting PCR library is sheared, ligated, and subjected to massively parallel clonal sequencing [86, 87]. However, like Roche 454, SOLiD™ and MethylSeq™ are based on emulsion PCR which can be troublesome and technically challenging.

The Illumina Solexa genome analyzer is the most widely used NGS strategy for DNA methylation analysis. It is based on in situ bridge template clonal amplification on a solid surface with amplicons remaining immobilized and clustered in a single physical location. Up to eight independent amplicon libraries are then sequenced in parallel using sequencing-by-synthesis technology that employs reversible terminators with removable fluorescent dyes. The Illumina Solexa genome analyzer can generate over 300 million reads per run at read lengths of up to 2 × 150 bp (http://www.illumina.com/systems/genome_analyzer_iix.ilmn). Both Applied Biosystems SOLiD™ and Illumina Solexa genome analyzer offer higher throughput and lower cost compared to Roche 454 but are more limited in alignment of bisulfite-converted sequences.

Other emerging single-molecule sequencing strategies bypass methylation-dependent treatments such as bisulfite modification prior to analysis. For example, two such new sequencing approaches are nano-sequencing and single-molecule, real-time (SMRT) sequencing [88, 89]. Nano-sequencing identifies methylation-based fluctuation in ionic current as DNA passes through a nanopore while SMRT-sequencing relies on emission spectra and polymerase kinetics during sequencing-by-synthesis for methylation analysis. These strategies offer the ability to perform highly sensitive methylation analyses of minute DNA quantities that is free of methylation-dependent treatment and amplification artifacts. Moreover, nano- and SMRT-sequencing have been shown to distinguish 5-mC from 5-hmC without any DNA pretreatments [90, 91].

#### Microarray-based DNA methylation profiling

In a technique, known as bisulfite methylation profiling (BiMP), bisulfite-treated DNA is subjected to whole genome amplification (WGA) using random tetranucleotide primers, enzymatic fragmentation, and microarray hybridization [92]. The microarray is designed using differentially labeled oligonucleotide pairs complementary to the unchanged, methylated sequence. Therefore, methylation is detected as a signal and mismatches caused by the conversion of unmethylated cytosines do not result in signal. This approach results in overall low hybridization signal and may not be applicable to regions of sparse methylation. The Infinium approach entails similar sample preparation that involves bisulfite modification of genomic DNA followed by WGA [93, 94]. The DNA is then hybridized to BeadChip microarrays, which are designed with oligonucleotide pairs targeting CpG sites of interest, with one complementary to the unchanged, methylated sequence and the other to the converted unmethylated sequence. Next, a PCR reaction is performed with fluorescently labeled universal PCR primers and the methylation levels can be determined by comparing the proportion of fluorescence emitted by each dye. Most microarray platforms contain a standard array of probes covering a library of CGIs. However, some companies also offer custom microarrays to allow for flexibility in experimental design and methylation analysis of CGI and/or organisms not available on standard microarrays. Furthermore, in the future era of personalized medicine, custom microarrays will be valuable for specific, individual methylation signatures.

### Microarray expression profiling

Genome-wide methylation profiling of samples representing diseased and normal state in search for biomarkers can be costly and time consuming. Therefore, some investigators prefer to narrow down the search using an expression-array following treatment with demethylating agents such as 5-aza-2′-deoxycytidine [95, 96]. This approach facilitates identification of genes that display evidence of methylation-dependent gene regulation in a disease state and understanding of disease pathobiology and progression. This approach identifies potential biomarkers, that is, those genes that are reactivated after the treatment. However, this strategy is prone to false results and is not considered to be a reliable measure of DNA methylation. This is because treatment with demethylating drugs alters the expression of many genes that (a) may not be related to disease state and (b) could stimulate expression of other, secondary targets. Therefore, methylation profiles of candidate biomarkers identified using this approach are further validated by other strategies.

## Validation of DNA Methylation-Based Biomarkers

**Figure 5:**
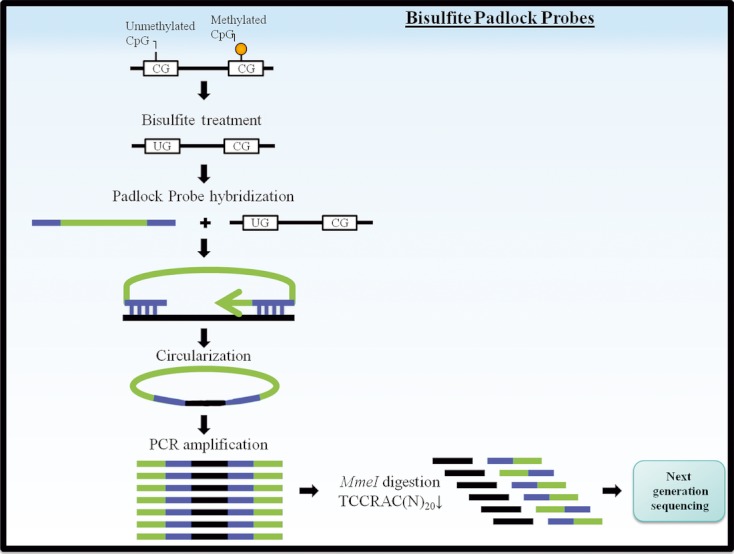
Schematic diagram of the bisulfite padlock probes approach to DNA methylation analysis. Bisulfite-modified DNA is combined with thousands of padlock probes that contain a common linker sequence represented in green. The library of padlock probes is hybridized to the bisulfite-converted DNA, circularized, and PCR amplified. The probes contain an enzyme digestion site such as *Mme*I-recognition site for uniform size selection. Next, the PCR-amplified DNA is digested and processed for next-generation bisulfite sequencing analysis.

Global DNA methylation screening approaches have their limitations and are prone to biases. Therefore, it is important to validate genome-wide assays with a quantitative, locus-specific assay, to assess quality and accuracy of the data and to determine whether specific methylation differences observed between samples are genuine. Majority of current gene-specific assays are PCR-based and are easily adapted to commercial platforms and can be used in clinical laboratories with high sensitivity and specificity [97]. Likewise, global 5-hmC detection strategies need to be validated using RE-, affinity- or bisulfite-based approaches combined with site specific, PCR-based platforms. Therefore, a number of methods have been developed to enrich for CpG harboring segments and survey a more limited region of the genome for methylation. One such method is bisulfite sequencing (BS) [79]. In this technique, genomic DNA is bisulfite modified and regions of interest are PCR amplified (Fig. 4). The PCR products are then cloned in *Escherichia coli* and numerous individual clones, each representing one PCR amplicon, are sequenced. The cloning step in this assay is necessary in order to isolate individual alleles, which differ in the pattern of methylated CpGs. However, it is costly, laborious, and time consuming. Therefore, recently digital PCR has been applied to BS [98]. Digital PCR is an alternative method for isolation of individual alleles which differ in methylation patterns. In digital PCR, the DNA sample is distributed over a 96-well PCR reaction plate so that individual DNA molecules are localized and amplified independently. The digital PCR products are purified and subjected to sequencing. BS is considered the gold-standard technique for DNA methylation analysis as it provides high-accuracy, single-nucleotide resolution information about the methylation status of almost any desired DNA segment. Therefore, BS has been extensively used to generate high-resolution maps of 5-mC in the CGI associated with a variety of promising biomarkers including *MGMT*, *CDKN*2A, and *MLH*1 to name a few [99–101]. More recently, strategies based on padlock probes have been developed as an alternative to enrichment for CpG-rich DNA fragments [102, 103]. Padlock probes consist of end segments, complementary to a target sequence, connected by a linker sequence. The end segments hybridize to bisulfite-converted target DNA in such a way that during ligation the probe becomes circularized around it. The linker sequence is then used for universal PCR allowing for the amplification of thousands of probes within a single reaction. The amplified targeted CpGs in padlock loops are then subjected to sequencing. In a technique called bisulfite padlock probes (BSPP), a library of padlock probes is hybridized to bisulfite-converted DNA, circularized, and PCR amplified. The resulting amplicons are then sequenced (Fig. 5). The main limitations of this method are sequence dependent bias of DNA polymerase and ligase, probe design, and hybridization efficiency.

Another strategy used to detect methylation in targeted DNA regions is pyrosequencing. Pyrosequencing is a “sequencing by synthesis” technique in which bisulfite-modified DNA is amplified using biotinylated primer (Fig. 4). The resulting biotin-labeled amplicons are denatured and utilized as a template for sequencing primers. During pyrosequencing, only one of the four nucleotides is present, and if incorporated into the sequence in a complementary base-pair wise manner, a pyrophosphate molecule is released as a reaction by-product. The release of pyrophosphate molecules is then quantitatively converted into a bioluminometric signal. Pyrosequencing has been widely used for methylation analysis in clinical specimens because it allows for direct quantitative sequencing of CpGs within a defined region of interest, accuracy, reproducibility, speed, and ease of use. Furthermore, the pyrosequencing technology has been incorporated into massively parallel sequencing on the 454 sequencing system to allow for genome-wide methylation analysis [104, 105].

An alternative sequencing platform for analysis of preselected CGIs is the GoldenGate assay, which has been previously reviewed by Chang et al. [106]. In this strategy, bisulfite-modified DNA undergoes allele-specific extension and ligation of specific CpG loci followed by PCR with universal primers and hybridization to bead microarrays.

**Figure 6:**
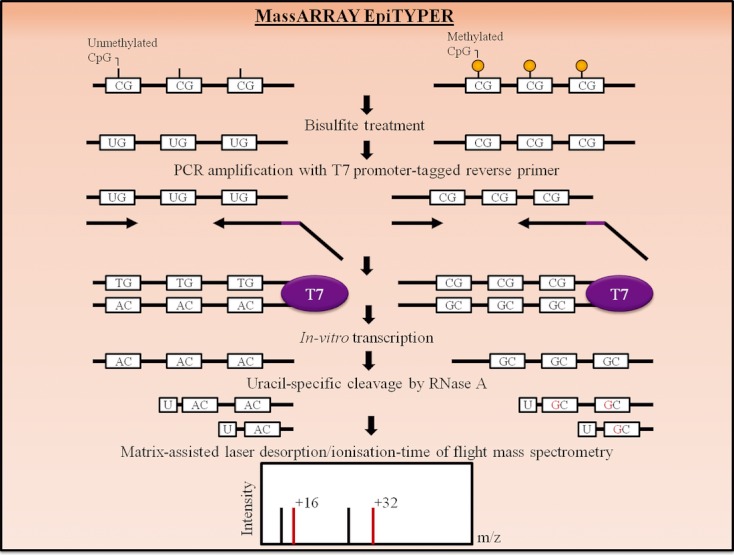
The basic principle of EpiTYPER analysis. Bisulfite-modified DNA is PCR amplified with T7 promoter-tagged reverse primer. Next, in vitro RNA transcription is performed, followed by digestion with RNase A. The digestion products are analyzed by MALDI-TOF MS. Methylated cytosines are transcribed to guanine, whereas unmethylated cytosines are converted to uracils and transcribed to adenines. This is represented in the mass spectrum by signal pairs separate by 16 m/z (or multiples thereof).

A more recent platform adapted for methylation analysis is matrix-assisted laser desorption ionization time-of-flight mass spectrometry (MALDI-TOF-MS). MassARRAY EpiTYPER assay uses this platform for quantitative base-specific methylation analysis of genomic regions of interest (Fig. 6) [107]. EpiTYPER can be used for biomarker discovery; however, the technology is especially well suited for precise sequencing using short DNA fragments and is more commonly used in candidate gene methylation analyses. In this assay, bisulfite-modified DNA amplicons with a T7-promoter tag are transcribed in vitro and digested with RNase A. Subsequently, the products are analyzed by MALDI-TOF-MS. Each C-to-T switch in the DNA following bisulfite conversion is identified on the MS as a mass difference of 16 Da. The main advantages of EpiTYPER are that it is fast, accurate, reproducible, and quantitative. However, some CpGs are missed by this technique when two fragments generated are of the exact same size, or fragments that are too small or too large to be analyzed. This technique has been previously used in our laboratory to provide accurate and quantitative methylation profiles of multiple CpGs in the Bone morphogenetic protein 7 (*BMP*7) and *HOXD*3 genes in prostate cancer samples [42]. Given that EpiTYPER analysis is based on bisulfite modification, it cannot differentiate 5-mC from 5-hmC. Alternative MS-based platforms can be used for specific 5-hmC quantification including HPLC-MS and liquid chromatography-MS [61, 108–110].

### Methylation-specific PCR (MSP) and quantitative variations of MSP

**Figure 7:**
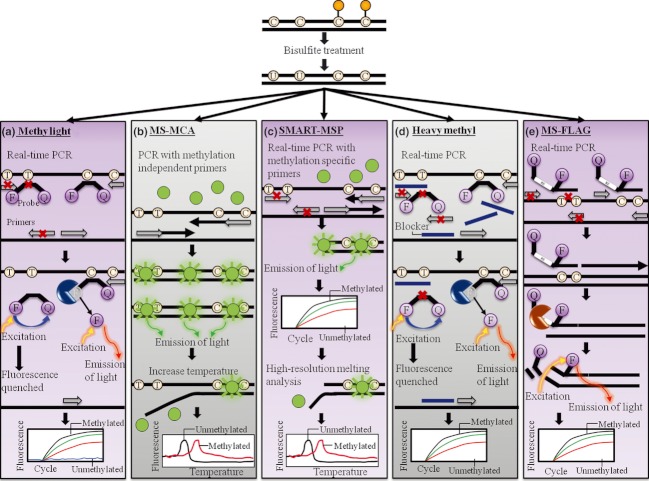
A panel of quantitative variations of methylation-specific PCR strategies including (a) MethyLight, (b) methylation-sensitive melting curve analysis (MS-MCA), (c) sensitive melting analysis after real-time SMART-MSP, (d) HeavyMethyl, and (e) methylation-specific fluorescent amplicon generation (MS-FLAG). (a) MethyLight utilizes methylation-specific primers and probe contains a fluorophore (F) and a quencher (Q) for specific amplification of methylated genomic DNA. During the PCR reaction, the probe is cleaved by the exonuclease activity of DNA polymerase, causing the fluorophore to be released from the quencher and light to be emitted. The emitted light signal is proportional to the amount of methylated DNA present in the sample. (b) In MS-MCA, bisulfite-treated DNA is PCR amplified with methylation-independent primers and double-stranded intercalating dye such as SYBR green (represented by green circles). Following PCR, the reaction temperature is increased and DNA melting properties are examined. Methylated DNA is C and G rich and consequently more resistant to melting. Therefore, more fluorescent signal is recorded at higher melting temperatures. (c) In SMART-MSP, bisulfite-modified DNA undergoes methylation-specific amplification in the presence of double-stranded intercalating dye such as SYBR green (represented by green circles) and the amount of signal detected is proportional to the amount of methylated DNA. Following PCR, the reaction temperature is increased and DNA melting properties are examined. (d) HeavyMethyl utilizes blocker oligonucleotides that specifically bind to unmethylated DNA and prevent its amplification. Alternatively, methylated DNA is amplified using methylation-independent primers and a methylation-specific probe that contains a fluorophore (F) and a quencher (Q). During the PCR reaction, the probe is cleaved by the exonuclease activity of DNA polymerase, causing the fluorophore to be released from the quencher and light to be emitted. The emitted light signal is proportional to the amount of methylated DNA present in the sample. (e) In MS-FLAG, bisulfite-treated DNA is amplified with methylation-specific primers that contain a cleavage site for *Psp*GI. Additionally, the primers contain a fluorophore (F) and a quencher (Q). The cleavage of the primers by *Psp*GI enables the release of the quencher from the fluorophore and light to be emitted, which is proportional to amount of methylated DNA.

MSP is the most widely used locus-specific bisulfite-based DNA methylation analysis strategy that has been reliably applied to a large scale of clinical samples and has been previously reviewed in the literature [10, 111]. Briefly, bisulfite-modified DNA serves as a template for PCR amplification using primer sets specific for methylated (MSP) and unmethylated (methylation-independent PCR) sequences. This is designed for proportional amplification of methylated and unmethylated DNA, respectively. MSP can also be coupled with in situ hybridization to visualize the methylation status of specific CpGs in individual cells [112]. It is a very popular technique because it is rapid, cost-effective, easy, and requires lesser quantities of DNA. However, it is prone to false positives, PCR contamination, and can only be used for qualitative analysis. Quantitative variations of this technique based on real-time PCR include MethyLight, methylation-sensitive melting curve analysis (MS-MCA), methylation-sensitive high-resolution melting (MS-HRM), sensitive melting analysis after real-time (SMART)-MSP, HeavyMethyl, and methylation-specific fluorescent amplicon generation (MS-FLAG) (Fig. 7), [113–118]. All these quantitative variations of MSP are highly sensitive real-time assays and are suitable for DNA methylation analysis of fresh, frozen, or formalin-fixed paraffin-embedded tissues and body fluid samples, such as serum, plasma, and urine.

MethyLight utilizes methylation-specific primers and a TaqMan methylation-specific fluorescent reporter probe that anneals to the amplified region of interest [113]. Annealing between the probe and methylated DNA results in fluorescent signal detection that is proportional to the amount of amplicon. Methylation levels are then determined by normalizing the signal to an Alu-based control reaction. MethyLight is a high-throughput, specific, sensitive, and quantitative assay that requires very small amounts of DNA; thus, it is suitable to be used in clinical laboratories. The utility of MethyLight for DNA methylation-based biomarker has been demonstrated by numerous studies, including the methylation of *GSTP*1, *APC*, *TGFβ*2, *HOXD*3, *MLH*1, dickkopf homolog 1(*DKK*1), and secreted frizzled-related protein 1 (*SFRP*1), which has been shown to be detected in prostate and colon cancers [43, 119–122]. More recently, MethyLight has been improved with the implementation of digital PCR [98]. However, this assay only allows quantitative methylation assessment of a few selected CpGs and is based on the assumption that all CpGs within the region probed share the same methylation status. Therefore, the selection of informative CpGs is crucial.

MS-MCA is a method that employs an intercalating double-stranded DNA fluorescent dye such as SYBR green to monitor the melting properties of PCR products during MSP as temperatures rise [114]. DNA melting curves are acquired by measuring the fluorescence during a linear temperature transition. Methylated DNA following bisulfite modification contains higher GC content, thus making it more resistant to melting. As a result, more fluorescent signal is recorded at higher melting temperatures. Methylation status of an unknown sample is then determined by comparing its melting profile with the melting profiles of controls obtained from the amplification of fully methylated and unmethylated molecules. MS-HRM is an improvement of MS-MCA that acquires more data points, thus allowing for subtle differences within the amplicons to be detected. More information about this strategy is available in a review by Kristensen et al. [111, 115]. Another method called SMART-MSP involves WGA, bisulfite modification, and probe-free real-time MSP with a fluorescent dye followed by HRM [116]. In this approach, methylation levels are determined based on fluorescent signal detection during MSP and melting profiles during HRM. Methylation levels are determined by normalization to a control assay such as collagen, type II, and alpha 1 (*COL*2A1) as well as to fully methylated and unmethylated standards. The limitations of all methods based on MCA are the use of dyes and the necessity of special equipment. Additionally, when heterogeneously methylated molecules are analyzed by MCA, the melting pattern becomes complex and difficult to interpret.

An alternative approach called HeavyMethyl uses methylation-independent primers and oligonucleotide blockers that hybridize only to unmethylated DNA [117]. Thus, only methylated DNA is amplified. The use of blockers to prevent unmethylated DNA amplification increases analytical sensitivity and reduces false-positive rate. This strategy also employs a fluorescent probe and fluorescent signal detection is used to quantify DNA methylation. Methylation status is quantified by normalization to a reference housekeeping gene such as β actin (ACTB) in a duplex PCR reaction. This is approach is more complicated than other approaches and requires a more accurate optimization. MS-FLAG is another quantitative MSP approach that relies on fluorescence [118]. In MS-FLAG, the real-time fluorescence signal is detected during PCR by cleavage of the MSP primers containing a fluorophore by a thermostable endonuclease. Methyl-BEAMing is a recently developed system based on methylation-independent PCR amplification of individual bisulfite-converted DNA molecules attached to magnetic beads within aqueous nano-compartments suspended in oil phase [123]. Following PCR, the beads are collected, incubated with fluorescent probes that specifically hybridize to methylated sequences, and analyzed using flow-cytometry. Methylation levels are then determined by normalization to long interspersed nuclear elements (LINE1)-based control reactions. This approach has been successfully applied to the analysis of vimentin methylation as a potential diagnostic biomarker for colorectal cancer [123].

### Methylation-sensitive single-nucleotide primer extension (MS-SNuPE)

MS-SNuPE is another bisulfite modification-based strategy that has been previously reviewed [124, 125]. The assay involves amplification of bisulfite-modified DNA with primers that terminate prior to the cytosine residue to be assayed. Next, on primer annealing, the primers are extended with radioactive nucleotides and the methylation is identified based on the sequence visualized by autoradiography. To avoid radioactive labeling, SNaPshot, HPLC, and MIRA platforms have been combined with MS-SNuPE [126–128].

### Combined bisulfite restriction analysis (COBRA)

COBRA is a well-established bisulfite-based method that relies on methylation-independent DNA amplification and digestion with *Bst*UI, an enzyme that cuts unmodified cytosines [129]. Methylation levels are established by the relative amounts of digested and undigested PCR products. COBRA is a low-throughput, nonquantitative technique that can only analyze CpGs present in enzymatic restriction sites. Furthermore, the method is relatively labor-intensive yet cost-effective. An improved protocol for COBRA, called Bio-COBRA, has been developed with a microfluidic platform for more high-throughput, accurate, and quantitative DNA methylation analysis [130].

### Methylation-sensitive arbitrarily primed PCR (MS-AP-PCR) and amplification of intermethylated sites (AIMS)

The most well-known locus-specific DNA methylation analysis techniques based on methylation-sensitive RE are MS-AP-PCR and AIMS [131–133]. In MS-AP-PCR, DNA is digested with *Msp*I or *Hpa*II, whereas in AIMS, it is digested with *Sma*I and *Xma*I. However, both techniques suffer from low-resolution and low-throughput, require high DNA quality and quantity, and utilize radioactive materials. Consequently, MS-AP-PCR and AIMS are rarely used for methylation analysis nowadays.

### MeDIP-PCR

An alternative approach to enrich for methylated DNA is affinity-based enrichment. One class of affinity-based strategies, called MeDIP-PCR, utilize bead-immobilized anti-5-methylcytosine antibodies [134]. Gene-specific DNA methylation is subsequently analyzed by PCR. Affinity-based methods allow for rapid and specific assessment of methylation changes in a gene-specific manner. They are easy to use and commercially available. However, the methods require high DNA input, have a potential for false-positive results due to unspecific binding to unmethylated DNA, and do not yield quantitative information.

### Combination of methylated-DNA precipitation and methylation-sensitive restriction enzymes (COMPARE-MS)

This method combines RE digestion with *Alu*I and *Hpa*II and MIRA to enrich for methylated DNA followed by quantitative real-time PCR (qPCR) for a more sensitive and specific methylation analysis than either approach alone [135]. However, the assay is complex, labor-intensive, and time consuming.

## Conclusions and Future Perspectives

The development of DNA methylation-based biomarkers is an emerging and exciting area of research that holds promise for potential applications in diverse clinical settings. This review focuses on a large number of techniques that have been developed for methylation analysis at global and gene-specific levels for DNA methylation-based biomarkers discovery and validation. It is important to note that a key intermediate step between discovery and validation is the analysis of the heterogeneity of methylation in gene promoters and identification of contextually meaningful CpG sites that mediate gene transcription. DNA methylation changes may cause quantitative transcriptional changes and/or may lead to qualitative transcriptional silencing. Upstream regulatory regions of many genes are known to harbor more than one promoter. These promoters may serve to regulate expression of specific transcripts thereby leading to generation and/or expression of alternate transcripts. Differential methylation of such promoters may be context dependent – that is, certain promoters are preferentially regulated via methylation in certain tissues or cell types. Alternately, methylation signals of promoters may change in response to surrounding environmental milieu. Better understanding of these aspects will provide important clues underlying association of specific biomarkers with disease biology. However, there are still many challenges to the effective implementation of DNA methylation-based biomarkers. For example, many methylation studies published to date have not accounted for the presence of hydroxymethylated DNA. The role(s) of 5-hydroxymethylation is distinct from 5-mC and is being elucidated. Recent studies suggest that 5-hmC may serve as an intermediate in direct DNA demethylation [136, 137]. It is present in mammalian DNA at physiologically relevant levels and aberrant hydroxymethylation may lead to disease. For example, 5-hmC is already implicated in carcinogenesis as it is significantly decreased in prostate, colon, and breast cancer compared with normal tissue [138]. Systematic investigation of the distribution and function of 5-hmC marks in various cellular contexts is necessary. No single 5-mC or 5-hmC detection strategy to date is superior to others, and there is much to be done in the field of epigenetic biomarker analysis strategies that will close the gap between biomarker discovery and clinical adaptation.

Earlier methylation analyses relied exclusively on BS, but this approach has many challenges. Subsequently, array-based profiling approaches were leading the field of DNA methylation-based biomarker discovery, but NGS-based approaches have quickly caught up and are likely to become the platform of choice in the near future. If the $1000 personal genome becomes a reality in the future, personal epigenome will be a reality soon to follow. One can envision in the era of personalized medicine, individual methylation “signatures” will be tested in a variety of minimally invasive samples. Although currently the identification of methylation “signatures” is focused mostly on cancer, future focus will be on other diseases, beyond cancer.

With respect to future frontiers in array-based platforms, the development of a triple microarray that will allow highly sensitive analysis of disease-related changes in DNA methylation, histone modifications, and microRNA expression simultaneously will provide new insights for more comprehensive epigenetic biomarker development.

New advancements in epigenetic technologies in the future will most likely drive the development of easy, noninvasive, cost-effective, high-throughput, highly sensitive, and specific epigenetic tests in the clinic.

## References

[1] Waddington CH (1942). The epigenotype. Endeavour.

[2] Jablonka E, Lamb MJ (2002). The changing concept of epigenetics. Ann. N. Y. Acad. Sci.

[3] Bird A, Taggart M, Frommer M, Miller OJ, Macleod D (1985). A fraction of the mouse genome that is derived from islands of nonmethylated, CpG-rich DNA. Cell.

[4] Gardiner-Garden M, Frommer M (1987). CpG islands in vertebrate genomes. J. Mol. Biol.

[5] Irizarry RA, Ladd-Acosta C, Wen B (2009). The human colon cancer methylome shows similar hypo- and hypermethylation at conserved tissue-specific CpG island shores. Nat. Genet.

[6] Doi A, Park IH, Wen B (2009). Differential methylation of tissue- and cancer-specific CpG island shores distinguishes human induced pluripotent stem cells, embryonic stem cells and fibroblasts. Nat. Genet.

[7] Holliday R, Pugh JE (1975). DNA modification mechanisms and gene activity during development. Science.

[8] Riggs AD (1975). X inactivation, differentiation, and DNA methylation. Cytogenet. Cell Genet.

[9] Haedicke W, Lesche R (2006). DNA methylation: potential for diagnosis, prognosis and therapy – prediction in oncology. Verh. Dtsch. Ges. Pathol.

[10] Herman JG, Graff JR, Myohanen S, Nelkin BD, Baylin SB (1996). Methylation-specific PCR: a novel PCR assay for methylation status of CpG islands. Proc. Natl. Acad. Sci. USA.

[11] Paluszczak J, Baer-Dubowska W (2006). Epigenetic diagnostics of cancer – the application of DNA methylation markers. J. Appl. Genet.

[12] Mikeska T (2010). CILDA. The implications of heterogeneous DNA methylation for the accurate quantification of methylation. Epigenomics.

[13] Ito S, Shen L, Dai Q (2011). Tet proteins can convert 5-methylcytosine to 5-formylcytosine and 5-carboxylcytosine. Science.

[14] Kriaucionis S, Heintz N (2009). The nuclear DNA base 5-hydroxymethylcytosine is present in Purkinje neurons and the brain. Science.

[15] Penn NW, Suwalski R, O'Riley C, Bojanowski K, Yura R (1972). The presence of 5-hydroxymethylcytosine in animal deoxyribonucleic acid. Biochem. J.

[16] Tahiliani M, Koh KP, Shen Y (2009). Conversion of 5-methylcytosine to 5-hydroxymethylcytosine in mammalian DNA by MLL partner TET1. Science.

[17] Haffner MC, Chaux A, Meeker AK (2011). Global 5-hydroxymethylcytosine content is significantly reduced in tissue stem/progenitor cell compartments and in human cancers. Oncotarget.

[18] Robertson J, Robertson AB, Klungland A (2011). The presence of 5-hydroxymethylcytosine at the gene promoter and not in the gene body negatively regulates gene expression. Biochem. Biophys. Res. Commun.

[19] Ficz G, Branco MR, Seisenberger S (2011). Dynamic regulation of 5-hydroxymethylcytosine in mouse ES cells and during differentiation. Nature.

[20] Xu Y, Wu F, Tan L (2011). Genome-wide regulation of 5hmC, 5mC, and gene expression by Tet1 hydroxylase in mouse embryonic stem cells. Mol. Cell.

[21] Yang H, Liu Y, Bai F (2012). Tumor development is associated with decrease of TET gene expression and 5-methylcytosine hydroxylation. Oncogene.

[22] Hayashizaki Y, Hirotsune S, Okazaki Y (1993). Restriction landmark genomic scanning method and its various applications. Electrophoresis.

[23] Hatada I, Hayashizaki Y, Hirotsune S, Komatsubara H, Mukai T (1991). A genomic scanning method for higher organisms using restriction sites as landmarks. Proc. Natl. Acad. Sci. USA.

[24] Smiraglia DJ, Plass C (2002). The study of aberrant methylation in cancer via restriction landmark genomic scanning. Oncogene.

[25] Khulan B, Thompson RF, Ye K (2006). Comparative isoschizomer profiling of cytosine methylation: the HELP assay. Genome Res.

[26] Oda M, Glass JL, Thompson RF (2009). High-resolution genome-wide cytosine methylation profiling with simultaneous copy number analysis and optimization for limited cell numbers. Nucleic Acids Res.

[27] Brunner AL, Johnson DS, Kim SW (2009). Distinct DNA methylation patterns characterize differentiated human embryonic stem cells and developing human fetal liver. Genome Res.

[28] Karimi M, Johansson S, Ekstrom TJ (2006). Using LUMA: a luminometric-based assay for global DNA-methylation. Epigenetics.

[29] Karimi M, Johansson S, Stach D (2006). LUMA (LUminometric Methylation Assay) – a high throughput method to the analysis of genomic DNA methylation. Exp. Cell Res.

[30] Ball MP, Li JB, Gao Y (2009). Targeted and genome-scale strategies reveal gene-body methylation signatures in human cells. Nat. Biotechnol.

[31] Toyota M, Ho C, Ahuja N (1999). Identification of differentially methylated sequences in colorectal cancer by methylated CpG island amplification. Cancer Res.

[32] Estecio MR, Yan PS, Ibrahim AE (2007). High-throughput methylation profiling by MCA coupled to CpG island microarray. Genome Res.

[33] Hatada I, Kato A, Morita S (2002). A microarray-based method for detecting methylated loci. J. Hum. Genet.

[34] Fukasawa M, Kimura M, Morita S (2006). Microarray analysis of promoter methylation in lung cancers. J. Hum. Genet.

[35] Huang YW, Huang TH, Wang LS (2010). Profiling DNA methylomes from microarray to genome-scale sequencing. Technol. Cancer Res. Treat.

[36] Sutherland E, Coe L, Raleigh EA (1992). McrBC: a multisubunit GTP-dependent restriction endonuclease. J. Mol. Biol.

[37] Irizarry RA, Ladd-Acosta C, Carvalho B (2008). Comprehensive high-throughput arrays for relative methylation (CHARM). Genome Res.

[38] Ibrahim AE, Thorne NP, Baird K (2006). MMASS: an optimized array-based method for assessing CpG island methylation. Nucleic Acids Res.

[39] Ordway JM, Bedell JA, Citek RW (2006). Comprehensive DNA methylation profiling in a human cancer genome identifies novel epigenetic targets. Carcinogenesis.

[40] Ordway JM, Budiman MA, Korshunova Y (2007). Identification of novel high-frequency DNA methylation changes in breast cancer. PLoS One.

[41] Huang TH, Perry MR, Laux DE (1999). Methylation profiling of CpG islands in human breast cancer cells. Hum. Mol. Genet.

[42] Kron K, Pethe V, Briollais L (2009). Discovery of novel hypermethylated genes in prostate cancer using genomic CpG island microarrays. PLoS One.

[43] Liu L, Kron KJ, Pethe VV (2011). Association of tissue promoter methylation levels of APC, TGFbeta2, HOXD3, and RASSF1A with prostate cancer progression. Int. J. Cancer.

[44] Kerkel K, Spadola A, Yuan E (2008). Genomic surveys by methylation-sensitive SNP analysis identify sequence-dependent allele-specific DNA methylation. Nat. Genet.

[45] Yuan E, Haghighi F, White S (2006). A single nucleotide polymorphism chip-based method for combined genetic and epigenetic profiling: validation in decitabine therapy and tumor/normal comparisons. Cancer Res.

[46] Kane MF, Loda M, Gaida GM (1997). Methylation of the hMLH1 promoter correlates with lack of expression of hMLH1 in sporadic colon tumors and mismatch repair-defective human tumor cell lines. Cancer Res.

[47] Lee WH, Morton RA, Epstein JI (1994). Cytidine methylation of regulatory sequences near the pi-class glutathione S-transferase gene accompanies human prostatic carcinogenesis. Proc. Natl. Acad. Sci. USA.

[48] Pieper RO, Costello JF, Kroes RA, Futscher BW, Marathi U, Erickson LC (1991). Direct correlation between methylation status and expression of the human O-6-methylguanine DNA methyltransferase gene. Cancer Commun.

[49] Nestor C, Ruzov A, Meehan R, Dunican D (2010). Enzymatic approaches and bisulfite sequencing cannot distinguish between 5-methylcytosine and 5-hydroxymethylcytosine in DNA. Biotechniques.

[50] Kinney SM, Chin HG, Vaisvila R (2011). Tissue-specific distribution and dynamic changes of 5-hydroxymethylcytosine in mammalian genomes. J. Biol. Chem.

[51] Janosi L, Yonemitsu H, Hong H, Kaji A (1994). Molecular cloning and expression of a novel hydroxymethylcytosine-specific restriction enzyme (PvuRts1I) modulated by glucosylation of DNA. J. Mol. Biol.

[52] Weber M, Davies JJ, Wittig D (2005). Chromosome-wide and promoter-specific analyses identify sites of differential DNA methylation in normal and transformed human cells. Nat. Genet.

[53] Down TA, Rakyan VK, Turner DJ (2008). A Bayesian deconvolution strategy for immunoprecipitation-based DNA methylome analysis. Nat. Biotechnol.

[54] Zilberman D, Gehring M, Tran RK, Ballinger T, Henikoff S (2007). Genome-wide analysis of *Arabidopsis thaliana* DNA methylation uncovers an interdependence between methylation and transcription. Nat. Genet.

[55] Zhang X, Yazaki J, Sundaresan A (2006). Genome-wide high-resolution mapping and functional analysis of DNA methylation in arabidopsis. Cell.

[56] Cross SH, Charlton JA, Nan X, Bird AP (1994). Purification of CpG islands using a methylated DNA binding column. Nat. Genet.

[57] Rauch T, Pfeifer GP (2005). Methylated-CpG island recovery assay: a new technique for the rapid detection of methylated-CpG islands in cancer. Lab. Invest.

[58] Gebhard C, Schwarzfischer L, Pham TH (2006). Genome-wide profiling of CpG methylation identifies novel targets of aberrant hypermethylation in myeloid leukemia. Cancer Res.

[59] Valinluck V, Tsai HH, Rogstad DK, Burdzy A, Bird A, Sowers LC (2004). Oxidative damage to methyl-CpG sequences inhibits the binding of the methyl-CpG binding domain (MBD) of methyl-CpG binding protein 2 (MeCP2). Nucleic Acids Res.

[60] Jin SG, Kadam S, Pfeifer GP (2010). Examination of the specificity of DNA methylation profiling techniques towards 5-methylcytosine and 5-hydroxymethylcytosine. Nucleic Acids Res.

[61] Globisch D, Munzel M, Muller M (2010). Tissue distribution of 5-hydroxymethylcytosine and search for active demethylation intermediates. PLoS One.

[62] Iqbal K, Jin SG, Pfeifer GP, Szabo PE (2011). Reprogramming of the paternal genome upon fertilization involves genome-wide oxidation of 5-methylcytosine. Proc. Natl. Acad. Sci. USA.

[63] Song CX, Szulwach KE, Fu Y (2010). Selective chemical labeling reveals the genome-wide distribution of 5-hydroxymethylcytosine. Nat. Biotechnol.

[64] Ko M, Huang Y, Jankowska AM (2010). Impaired hydroxylation of 5-methylcytosine in myeloid cancers with mutant TET2. Nature.

[65] Robertson AB, Dahl JA, Vagbo CB, Tripathi P, Krokan HE, Klungland A (2011). A novel method for the efficient and selective identification of 5-hydroxymethylcytosine in genomic DNA. Nucleic Acids Res.

[66] Hayatsu H, Wataya Y, Kai K, Iida S (1970). Reaction of sodium bisulfite with uracil, cytosine, and their derivatives. Biochemistry.

[67] Huang Y, Pastor WA, Shen Y, Tahiliani M, Liu DR, Rao A (2010). The behaviour of 5-hydroxymethylcytosine in bisulfite sequencing. PLoS One.

[68] Booth MJ, Branco MR, Ficz G (2012). Quantitative sequencing of 5-methylcytosine and 5-hydroxymethylcytosine at single-base resolution. Science.

[69] Pastor WA, Pape UJ, Huang Y (2011). Genome-wide mapping of 5-hydroxymethylcytosine in embryonic stem cells. Nature.

[70] Szwagierczak A, Bultmann S, Schmidt CS, Spada F, Leonhardt H (2010). Sensitive enzymatic quantification of 5-hydroxymethylcytosine in genomic DNA. Nucleic Acids Res.

[71] Liutkeviciute Z, Kriukiene E, Grigaityte I, Masevicius V, Klimasauskas S (2011). Methyltransferase-directed derivatization of 5-hydroxymethylcytosine in DNA. Angew. Chem. Int. Ed. Engl.

[72] Bormann Chung CA, Boyd VL, McKernan KJ (2012). Whole methylome analysis by ultra-deep sequencing using two-base encoding. PLoS One.

[73] Lister R, Pelizzola M, Dowen RH (2009). Human DNA methylomes at base resolution show widespread epigenomic differences. Nature.

[74] Cokus SJ, Feng S, Zhang X (2008). Shotgun bisulphite sequencing of the Arabidopsis genome reveals DNA methylation patterning. Nature.

[75] Lister R, O'Malley RC, Tonti-Filippini J (2008). Highly integrated single-base resolution maps of the epigenome in Arabidopsis. Cell.

[76] Meissner A, Gnirke A, Bell GW, Ramsahoye B, Lander ES, Jaenisch R (2005). Reduced representation bisulfite sequencing for comparative high-resolution DNA methylation analysis. Nucleic Acids Res.

[77] Meissner A, Mikkelsen TS, Gu H (2008). Genome-scale DNA methylation maps of pluripotent and differentiated cells. Nature.

[78] Baumer A, Wiedemann U, Hergersberg M, Schinzel A (2001). A novel MSP/DHPLC method for the investigation of the methylation status of imprinted genes enables the molecular detection of low cell mosaicisms. Hum. Mutat.

[79] Frommer M, McDonald LE, Millar DS (1992). A genomic sequencing protocol that yields a positive display of 5-methylcytosine residues in individual DNA strands. Proc. Natl. Acad. Sci. USA.

[80] Taylor KH, Kramer RS, Davis JW (2007). Ultradeep bisulfite sequencing analysis of DNA methylation patterns in multiple gene promoters by 454 sequencing. Cancer Res.

[81] Margulies M, Egholm M, Altman WE (2005). Genome sequencing in microfabricated high-density picolitre reactors. Nature.

[82] Valouev A, Ichikawa J, Tonthat T (2008). A high-resolution, nucleosome position map of *C. elegans* reveals a lack of universal sequence-dictated positioning. Genome Res.

[83] Bentley DR, Balasubramanian S, Swerdlow HP (2008). Accurate whole human genome sequencing using reversible terminator chemistry. Nature.

[84] Shendure J, Porreca GJ, Reppas NB (2005). Accurate multiplex polony sequencing of an evolved bacterial genome. Science.

[85] Milos P (2008). Helicos BioSciences. Pharmacogenomics.

[86] Herrmann A, Haake A, Ammerpohl O (2011). Pipeline for large-scale microdroplet bisulfite pcr-based sequencing allows the tracking of hepitype evolution in tumors. PLoS One.

[87] Komori HK, Lamere SA, Torkamani A (2011). Application of microdroplet PCR for large-scale targeted bisulfite sequencing. Genome Res.

[88] Clarke J, Wu HC, Jayasinghe L, Patel A, Reid S, Bayley H (2009). Continuous base identification for single-molecule nanopore DNA sequencing. Nat. Nanotechnol.

[89] Flusberg BA, Webster DR, Lee JH (2000). Direct detection of DNA methylation during single-molecule, real-time sequencing. Nat. Methods.

[90] Wallace EV, Stoddart D, Heron AJ (2010). Identification of epigenetic DNA modifications with a protein nanopore. Chem. Commun. (Camb.).

[91] Flusberg BA, Webster DR, Lee JH (2010). Direct detection of DNA methylation during single-molecule, real-time sequencing. Nat. Methods.

[92] Reinders J, Delucinge Vivier C, Theiler G, Chollet D, Descombes P, Paszkowski J (2008). Genome-wide, high-resolution DNA methylation profiling using bisulfite-mediated cytosine conversion. Genome Res.

[93] Bibikova M, Lin Z, Zhou L (2006). High-throughput DNA methylation profiling using universal bead arrays. Genome Res.

[94] Weisenberger DJ, Van Den Berg D, Pan F, Berman BP, Laird PW (2008). Comprehensive DNA methylation analysis on the Illumina® Infinium® assay platform.

[95] Suzuki H, Gabrielson E, Chen W (2002). A genomic screen for genes upregulated by demethylation and histone deacetylase inhibition in human colorectal cancer. Nat. Genet.

[96] Jones PA, Taylor SM (1980). Cellular differentiation, cytidine analogs and DNA methylation. Cell.

[97] Houghton SG, Cockerill FR (2006). Real-time PCR: overview and applications. Surgery.

[98] Weisenberger DJ, Trinh BN, Campan M (2008). DNA methylation analysis by digital bisulfite genomic sequencing and digital MethyLight. Nucleic Acids Res.

[99] Watts GS, Pieper RO, Costello JF, Peng YM, Dalton WS, Futscher BW (1997). Methylation of discrete regions of the O6-methylguanine DNA methyltransferase (MGMT) CpG island is associated with heterochromatinization of the MGMT transcription start site and silencing of the gene. Mol. Cell. Biol.

[100] Gonzalgo ML, Hayashida T, Bender CM (1998). The role of DNA methylation in expression of the p19/p16 locus in human bladder cancer cell lines. Cancer Res.

[101] Deng G, Chen A, Hong J, Chae HS, Kim YS (1999). Methylation of CpG in a small region of the hMLH1 promoter invariably correlates with the absence of gene expression. Cancer Res.

[102] Deng J, Shoemaker R, Xie B (2009). Targeted bisulfite sequencing reveals changes in DNA methylation associated with nuclear reprogramming. Nat. Biotechnol.

[103] Porreca GJ, Zhang K, Li JB (2007). Multiplex amplification of large sets of human exons. Nat. Methods.

[104] Colella S, Shen L, Baggerly KA, Issa JP, Krahe R (2003). Sensitive and quantitative universal pyrosequencing methylation analysis of CpG sites. Biotechniques.

[105] Tost J, Dunker J, Gut IG (2003). Analysis and quantification of multiple methylation variable positions in cpg islands by pyrosequencing. Biotechniques.

[106] Chang JW, Huang TH, Wang YC (2008). Emerging methods for analysis of the cancer methylome. Pharmacogenomics.

[107] Ehrich M, Nelson MR, Stanssens P (2005). Quantitative high-throughput analysis of DNA methylation patterns by base-specific cleavage and mass spectrometry. Proc. Natl. Acad. Sci. USA.

[108] Burdzy A, Noyes KT, Valinluck V, Sowers LC (2002). Synthesis of stable-isotope enriched 5-methylpyrimidines and their use as probes of base reactivity in DNA. Nucleic Acids Res.

[109] Feng J, Zhou Y, Campbell SL (2010). Dnmt1 and Dnmt3a maintain DNA methylation and regulate synaptic function in adult forebrain neurons. Nat. Neurosci.

[110] Le T, Kim KP, Fan G, Faull KF (2011). A sensitive mass spectrometry method for simultaneous quantification of DNA methylation and hydroxymethylation levels in biological samples. Anal. Biochem.

[111] Kristensen LS, Hansen LL (2009). PCR-based methods for detecting single-locus DNA methylation biomarkers in cancer diagnostics, prognostics, and response to treatment. Clin. Chem.

[112] Nuovo GJ, Plaia TW, Belinsky SA, Baylin SB, Herman JG (1999). In situ detection of the hypermethylation-induced inactivation of the p16 gene as an early event in oncogenesis. Proc. Natl. Acad. Sci. USA.

[113] Eads CA, Danenberg KD, Kawakami K (2000). MethyLight: a high-throughput assay to measure DNA methylation. Nucleic Acids Res.

[114] Worm J, Aggerholm A, Guldberg P (2001). In-tube DNA methylation profiling by fluorescence melting curve analysis. Clin. Chem.

[115] Wojdacz TK, Dobrovic A (2007). Methylation-sensitive high resolution melting (MS-HRM): a new approach for sensitive and high-throughput assessment of methylation. Nucleic Acids Res.

[116] Kristensen LS, Mikeska T, Krypuy M, Dobrovic A (2008). Sensitive melting analysis after real time- methylation specific PCR (SMART-MSP): high-throughput and probe-free quantitative DNA methylation detection. Nucleic Acids Res.

[117] Cottrell SE, Distler J, Goodman NS (2004). A real-time PCR assay for DNA-methylation using methylation-specific blockers. Nucleic Acids Res.

[118] Bonanno C, Shehi E, Adlerstein D, Makrigiorgos GM (2007). MS-FLAG, a novel real-time signal generation method for methylation-specific PCR. Clin. Chem.

[119] Kron KJ, Liu L, Pethe VV (2010). DNA methylation of HOXD3 as a marker of prostate cancer progression. Lab. Invest.

[120] Matuschek C, Bolke E, Lammering G (2010). Methylated APC and GSTP1 genes in serum DNA correlate with the presence of circulating blood tumor cells and are associated with a more aggressive and advanced breast cancer disease. Eur. J. Med. Res.

[121] Mrkonjic M, Roslin NM, Greenwood CM (2010). Specific variants in the MLH1 gene region may drive DNA methylation, loss of protein expression, and MSI-H colorectal cancer. PLoS One.

[122] Rawson JB, Manno M, Mrkonjic M (2011). Promoter methylation of Wnt antagonists DKK1 and SFRP1 are associated with opposing tumor subtypes in a large cohort of colorectal cancers. Carcinogenesis.

[123] Li M, Chen WD, Papadopoulos N (2009). Sensitive digital quantification of DNA methylation in clinical samples. Nat. Biotechnol.

[124] Gonzalgo ML, Jones PA (1997). Rapid quantitation of methylation differences at specific sites using methylation-sensitive single nucleotide primer extension (Ms-SNuPE). Nucleic Acids Res.

[125] Fraga MF, Esteller M (2002). DNA methylation: a profile of methods and applications. Biotechniques.

[126] El-Maarri O, Herbiniaux U, Walter J, Oldenburg J (2002). A rapid, quantitative, non-radioactive bisulfite-SNuPE- IP RP HPLC assay for methylation analysis at specific CpG sites. Nucleic Acids Res.

[127] Kaminsky ZA, Assadzadeh A, Flanagan J, Petronis A (2005). Single nucleotide extension technology for quantitative site-specific evaluation of metC/C in GC-rich regions. Nucleic Acids Res.

[128] Lee DH, Tran DA, Singh P (2011). MIRA-SNuPE, a quantitative, multiplex method for measuring allele-specific DNA methylation. Epigenetics.

[129] Xiong Z, Laird PW (1997). COBRA: a sensitive and quantitative DNA methylation assay. Nucleic Acids Res.

[130] Brena RM, Auer H, Kornacker K, Plass C (2006). Quantification of DNA methylation in electrofluidics chips (Bio-COBRA). Nat. Protoc.

[131] Frigola J, Ribas M, Risques RA, Peinado MA (2002). Methylome profiling of cancer cells by amplification of inter-methylated sites (AIMS). Nucleic Acids Res.

[132] Gonzalgo ML, Liang G, Spruck CH, Zingg JM, Rideout WM, Jones PA (1997). Identification and characterization of differentially methylated regions of genomic DNA by methylation-sensitive arbitrarily primed PCR. Cancer Res.

[133] Huang TH, Laux DE, Hamlin BC, Tran P, Tran H, Lubahn DB (1997). Identification of DNA methylation markers for human breast carcinomas using the methylation-sensitive restriction fingerprinting technique. Cancer Res.

[134] Weber M, Hellmann I, Stadler MB (2007). Distribution, silencing potential and evolutionary impact of promoter DNA methylation in the human genome. Nat. Genet.

[135] Yegnasubramanian S, Lin X, Haffner MC, DeMarzo AM, Nelson WG (2006). Combination of methylated-DNA precipitation and methylation-sensitive restriction enzymes (COMPARE-MS) for the rapid, sensitive and quantitative detection of DNA methylation. Nucleic Acids Res.

[136] Guo JU, Su Y, Zhong C, Ming GL, Song H (2011). Hydroxylation of 5-methylcytosine by TET1 promotes active DNA demethylation in the adult brain. Cell.

[137] Williams K, Christensen J, Pedersen MT (2011). TET1 and hydroxymethylcytosine in transcription and DNA methylation fidelity. Nature.

[138] Haffner MC, Chaux A, Meeker AK (2011). Global 5-hydroxymethylcytosine content is significantly reduced in tissue stem/progenitor cell compartments and in human cancers. Oncotarget.

